# Ageing and BMI in Focus: Rethinking Risk Assessment for Vertebral Fragility and Pedicle Screw Loosening in Older Adults

**DOI:** 10.3390/jcm14155296

**Published:** 2025-07-27

**Authors:** Jun Li, André Strahl, Beate Kunze, Stefan Krebs, Martin Stangenberg, Lennart Viezens, Patrick Strube, Marc Dreimann

**Affiliations:** 1 Spine Center for Neuroorthopaedics, Spinal Cord Injuries, and Scoliosis, RKH Orthopaedic Clinic Markgröningen, 71706 Markgröningen, Germany; 2 Department of Psychosomatic Medicine and Psychotherapy, Centre for Internal Medicine, University Medical Center Hamburg-Eppendorf, 20246 Hamburg, Germany; 3 Department of Spinal Surgery and Neurosurgery, Tabea Hospital Hamburg, 22587 Hamburg, Germany; 4 Department of Trauma and Orthopaedic Surgery, Division of Spine Surgery, University Medical Center Hamburg-Eppendorf, 20246 Hamburg, Germany; 5 Department of Spine, German Center for Orthopaedics, Waldkliniken Eisenberg, 07607 Eisenberg, Germany

**Keywords:** vertebral fragility, pedicle screw loosening, ageing population, body mass index (BMI), posterior instrumented spinal fusion, spine surgery

## Abstract

**Background/Objectives**: Pathological vertebral fragility (path-VF) increases the risk of osteoporotic fractures and pedicle screw loosening (PSL) after posterior instrumented spinal fusion (PISF). While WHO body mass index (BMI) categories broadly identify risks related to underweight and obesity, fixed thresholds may inadequately reflect vertebral fragility risks among elderly patients, especially within the normal-weight range. This study investigates whether current BMI classifications sufficiently capture the risk of path-VF in older adults. **Methods**: This retrospective study included 225 patients who underwent kyphoplasty or PISF (2022–2023). Path-VF was defined by non-tumorous fractures, screw reinforcement, or PSL within six months without prior reinforcement. Patients were grouped into the path-VF (n = 94) and control (n = 131) groups. HU and BMI values, BMI-related ORs, and age trends were analysed, and a logistic regression was performed. **Results**: Mean HU values were significantly lower in the path-VF group (71.37 ± 30.50) than in controls (130.35 ± 52.53, *p* < 0.001). Path-VF females (26.26 ± 5.38) had a lower BMI than the control females (29.33 ± 5.98, *p* = 0.002); no difference was found in males. Normal-weight females showed a borderline risk for path-VF (OR 2.03, *p* = 0.0495). Obesity (OR_male_ 0.31/OR_female_ 0.37) and being male and overweight (OR 0.21) were protective (all *p* < 0.05). BMI declined with age in path-VF males (*p* = 0.001) but increased in the controls (*p* = 0.023). A logistic regression identified a BMI < 22.5 kg/m^2^ and age > 67.5 years as significant risk thresholds. Notably, 20.2% of path-VF patients over 67.5 had a normal weight, suggesting a potentially overlooked subgroup. **Conclusions**: The current WHO lower limit for normal BMI (18.5 kg/m^2^) may underestimate the risk of path-VF in patients older than 67.5 years, potentially overlooking 24.7% of cases. The results offer a new approach for clinicians to interpret BMI values at the lower end of the normal range (<22.5 kg/m^2^) with caution in elderly patients undergoing spinal surgery.

## 1. Introduction

Vertebral fragility (VF), characterised by reduced vertebral strength [1], significantly increases the risk of pathological fractures and early pedicle screw loosening (PSL) following posterior instrumented spinal fusion (PISF), even under normal physiological loading conditions [2]. VF, closely linked to osteoporosis and osteoporotic fractures [3], may progress asymptomatically and is influenced by multiple factors, including advanced age, obesity, previous fragility fractures, and anti-osteoporosis medications [4].

Successful outcomes after thoracic and lumbar PISF depend largely on vertebral biomechanics and patient-related factors, such as age, gender, nutrition, lifestyle, and body mass index (BMI) [5,6,7]. Although bone density measurement is standard for assessing VF, it is frequently omitted during preoperative planning [8]. Instead, the World Health Organisation (WHO) BMI classification is commonly applied to broadly evaluate nutritional status and surgical risk [9].

In spinal surgery, obesity and overweight status increase risks for conditions, such as spondylitis [10], and negatively impact outcomes by prolonging surgery duration, increasing blood loss, infection rates, and delayed healing [11,12,13]. Similarly, underweight patients often face complications related to malnutrition, poor bone quality, and reduced muscle mass, elevating the risk of hardware failure and impaired recovery [6,14,15]. Poor BMD, common among underweight individuals, also diminishes bone anchoring capacity and increases fracture susceptibility [16,17,18].

However, limited research has evaluated whether WHO BMI categories reliably predict postoperative complications specifically in ageing populations. Winter et al. [19] reported significantly increased mortality in elderly individuals (aged > 65) at a BMI < 23, while an elevated mortality among obese elderly individuals was seen only above a BMI of 33. Such findings raise questions about whether static BMI thresholds adequately reflect the nuanced health risks in elderly spinal surgery patients, particularly regarding pathological vertebral fragility.

To our knowledge, no prior research has specifically investigated whether the current WHO BMI classification accurately reflects the risk of pathological vertebral fragility (path-VF) in elderly patients, particularly whether a BMI within the ‘normal weight’ range of 18.5 to 24.9 kg/m^2^ truly indicates no elevated risk. This study aims to address this gap.

## 2. Materials and Methods

### 2.1. Patients

This retrospective case-control study evaluated 225 patients treated at a single spine centre between 2022 and 2023, all of whom underwent their first balloon kyphoplasty or posterior instrumented spinal fusion (PISF) of the thoracic or lumbar spine. Preoperative demographic and BMI data were collected.

### 2.2. Inclusion and Exclusion Criteria

Inclusion Criteria:

Patients were included in the study if they presented with pathological vertebral fragility (path-VF), defined by non-tumorous pathological vertebral fractures necessitating balloon kyphoplasty, the requirement for intraoperative pedicle screw reinforcement during initial posterior instrumented spinal fusion (PISF), or non-traumatic pedicle screw loosening (PSL) within six months post-PISF without prior screw reinforcement. Both male and female patients were eligible, and no age limits were imposed for inclusion; however, while most participants were adults, individuals under 18 years old were only included if their surgical indication was severe spinal deformity (idiopathic scoliosis) and they had achieved full skeletal maturity at the time of surgery.

The path-VF group was defined based on a shared insufficiency of vertebral strength, resulting from factors such as low BMD, malnutrition, sarcopenia, and age-related decline [1,20]. The clinical manifestations ranged from fracture and screw reinforcement to early loosening, and the degree of fragility spanned from osteopenia to established osteoporosis, reflecting a spectrum of severity.

Surgical indications for PISF included the following:

(1) Severe degenerative spinal stenosis with instability;
(2) Idiopathic scoliosis;
(3) Spondylolisthesis;
(4) De novo lumbar scoliosis.

Exclusion Criteria:

Patients were excluded from the study if they had clinically suspected or confirmed spondylodiscitis, or if they presented with primary spinal tumours or spinal metastases originating from other malignancies.

### 2.3. Methodological Details of Screw Augmentation and Loosening Diagnosis

In this study, only cement-augmented screws were utilized, as this is the most established method for screw reinforcement; other techniques, such as expandable screws or bioactive coatings, were not included [21,22]. While subjective assessment might miss up to 31% of screws requiring augmentation in cases of low BMD, no overtreatment occurred in vertebrae with normal BMD, suggesting a low risk of overlooking true fragility [8]. A six-month threshold was applied to identify PSL related to bone fragility, thereby excluding later cases more likely attributable to mechanical overload or infection [23]. The diagnosis of PSL was confirmed via CT scans demonstrating a radiolucent rim greater than 1 mm or evidence of screw pull-out/cutting out [24]; these scans were performed only when postoperative symptoms, such as severe pain or limited mobility, indicated potential complications [25]. Finally, screw length was selected to reach the anterior third of the vertebral body without cortical breach, and the diameter was chosen to match the pedicle size for optimal fixation.

### 2.4. Measurement of Vertebral Hounsfield Unit (HU) Values

Given the strong correlation between vertebral HU values, BMD, and pedicle screw fixation stability [2,26], we retrospectively analysed mean HU values from vertebrae T11 to L5 on preoperative lumbar CT scans in 99 patients to validate risk grouping relative to bone quality. HU values were measured in elliptical regions of cancellous bone at the cephalad (middle) junction of each vertebral body from T11 to L5, excluding cortical bone, vascular tissue, Schmorl’s nodes, cystic changes, severe endplate sclerosis, fractures, and tumours. Mean values across T11–L5 were utilised for statistical analysis.

### 2.5. Grouping and BMI Categories

Patients were classified into two groups: the path-VF group and those without (control group). According to the WHO BMI criteria, patients were divided into four weight categories: underweight (BMI < 18.5 kg/m^2^), normal weight (BMI 18.5–24.9 kg/m^2^), overweight (BMI 25.0–29.9 kg/m^2^), and obese (BMI ≥ 30 kg/m^2^) [9].

### 2.6. Statistical Analyses

Statistical analyses were performed using R software (version 4.4.1, The R Foundation for Statistical Computing, Vienna, Austria). Continuous variables are presented as mean ± standard deviation (SD), and statistical significance was defined as *p* < 0.05.

Student’s *t*-test or Mann–Whitney U-test was used to compare age, BMI, and HU values between path-VF and control groups, depending on normality and variance homogeneity assessments. The Shapiro–Wilk test and the Kolmogorov–Smirnov test were used to assess normality, while Levene’s test, Bartlett’s test, and the F-test were applied to evaluate homogeneity of variances. Effect sizes were assessed using Cohen’s *d* (*d*) for *t*-tests and rank-biserial correlation coefficient (*r*) for U-test. Thresholds for medium and large effects were set at >0.5 and >0.8 for *d* and >0.3 and >0.5 for *r*, respectively. Fisher’s exact test calculated odds ratios (ORs) with 95% confidence intervals (CIs) for BMI categories associated with the occurrence of path-VF. Pearson’s correlation test (coefficient: *r_p_*) and Fisher’s Z-test (coefficient Cohen’s *q*: *q*) were used to examine BMI–age correlations between the path-VF and control groups. Additionally, binary logistic regression and receiver operating characteristic (ROC) curve analyses were conducted to evaluate the influence of gender, age, and BMI on the occurrence of path-VF and to identify threshold values for age and BMI. The area under the curve (AUC) was interpreted as fair if 0.7 ≤ AUC < 0.8 and excellent if AUC ≥ 0.8 [27].

### 2.7. AI Statement

During the preparation of this manuscript, Trinka AI was used to assist with grammar and language editing. The corresponding author reviewed and revised the content afterward and takes full responsibility for the final version of the manuscript.

## 3. Results

### 3.1. Demographics of the Study Cohort

The study cohort consisted of 88 male and 137 female patients with an average age of 68.11 years (range: 17–92 years). One patient aged 17.28 years was not excluded due to severe scoliosis and confirmed skeletal maturity at the time of surgery. Treatment was consistent with adult protocols.

Detailed demographics are shown in Table 1.

Patients in the path-VF group (all aged >50 years) were significantly older than those in the control group, for both males (*p* < 0.001, *d* = 0.90) and females (*p* < 0.001, *d* = 1.01). However, within the path-VF group, no significant age difference was observed between genders (*p* > 0.05, *d* = 0.01).

The distribution of BMI categories across the path-VF and control groups by gender is detailed in Table 2. Notably, the number of underweight patients was very small for both genders.

### 3.2. Differences in Vertebral HU Values Between the Path-VF and Control Groups

**Figure 1:**
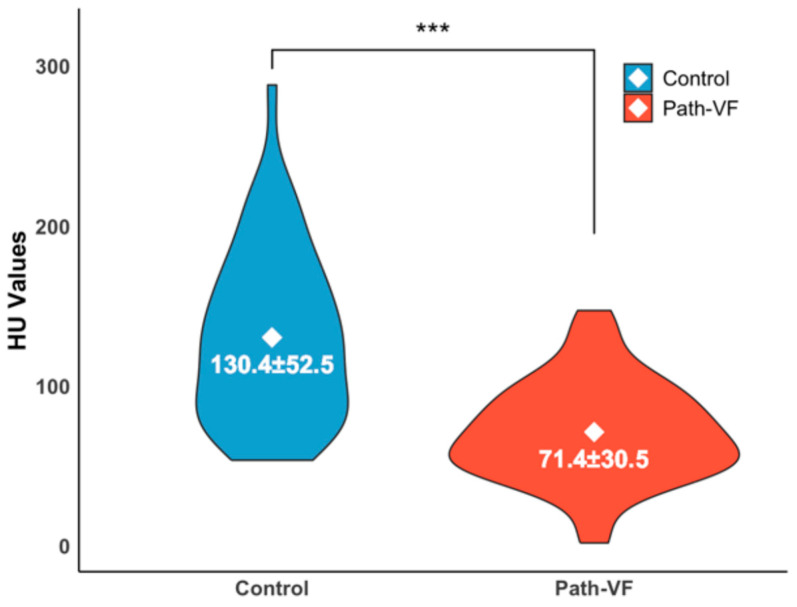
Violin plot of HU values of the path-VF and control groups. Mean ± SD, *** *p* < 0.001.

As illustrated in Figure 1, the mean HU value from vertebrae Th11 to L5 was significantly lower in the path-VF group (n = 52) compared to the control group (n = 47) (71.37 ± 30.50 vs. 130.35 ± 52.53, *p* < 0.001, *r* = 0.58), indicating reduced vertebral bone quality among patients with path-VF.

### 3.3. BMI Differences and Associations Between the Path-VF and Control Groups

**Figure 2:**
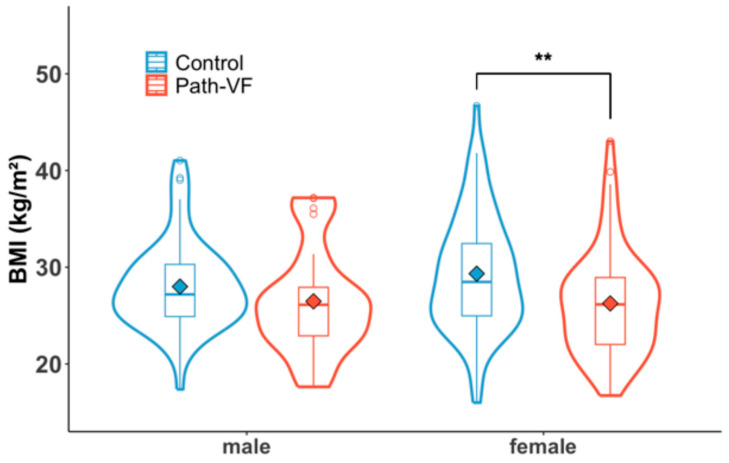
BMI of the path-VF and control groups in males and females. ** *p* < 0.01.

In males, BMI differences between the path-VF (26.47 ± 5.46) and control (28.00 ± 4.52) groups were not significant (*p* = 0.22, *d* = 0.32). Conversely, females in the path-VF group had significantly lower BMIs (26.26 ± 5.38) compared to the control group (29.33 ± 5.98; *p* = 0.002, *d* = 0.54). The BMI distributions in the violin plot (Figure 2) suggest a shift toward lower BMI ranges in the path-VF group for both genders; however, this difference reached statistical significance only in females.

**Figure 3:**
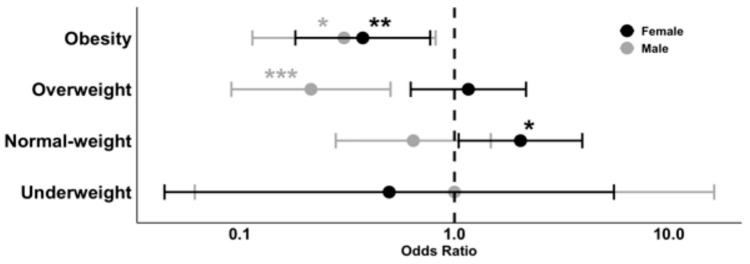
OR of BMI categories for the risk of path-V. * *p* < 0.05, ** *p* < 0.01, *** *p* < 0.001.

Obese males showed a significantly reduced risk of developing path-VF, with an OR of 0.31 (95% CI [0.11–0.82], *p* = 0.024). Being obese and female also demonstrated a protective effect, with an OR of 0.37 (95% CI [0.18–0.77], *p* = 0.009). In addition, overweight males had an even lower risk, with an OR of 0.21 (95% CI [0.09–0.50], *p* < 0.001). In contrast, normal-weight females were at significantly higher risk of path-VF, with an OR of 2.03 (95% CI [1.05–3.94], *p* = 0.0495) (Figure 3).

### 3.4. Age-Related Distributions of BMI

**Figure 4:**
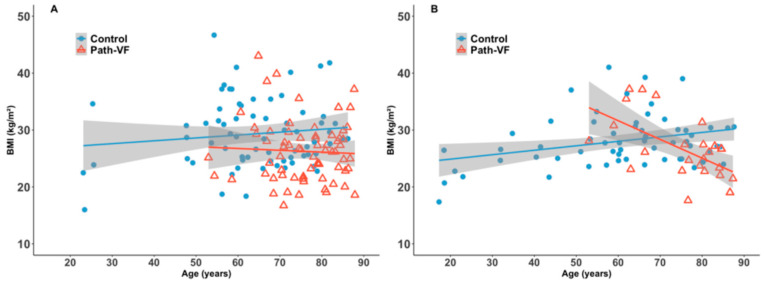
BMI distributions of the path-VF and control groups across ages. (**A**) Female patients; (**B**) male patients.

Furthermore, we explored the distribution trend of BMI in the path-VF and control groups with increasing age. In females (Figure 4A), no significant BMI–age correlations were observed (path-VF: *r_p_* = −0.05, *p* = 0.68; control: *r_p_* = 0.12, *p* = 0.33), and no crossover trend occurred (*p* = 0.33). Conversely, males (Figure 4B) showed significant BMI–age trends in the opposite direction: a significantly negative correlation in the path-VF group (*r_p_* = −0.59, *p* = 0.001) and a positive correlation in the control group (*r_p_* = 0.29, *p* = 0.02), with a significant crossover effect around age 70 (*p* < 0.001, *q* = −0.98).

**Figure 5:**
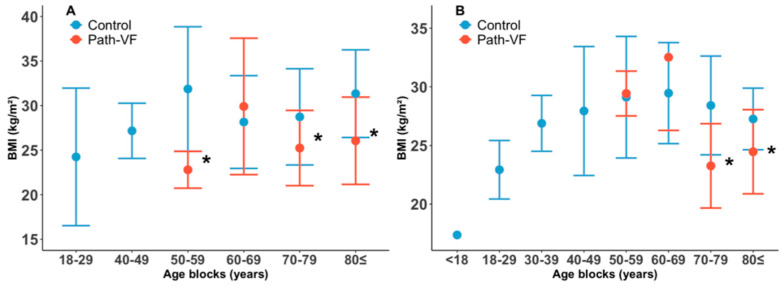
BMIs across age blocks of the path-VF and control groups. (**A**) Female patients; (**B**) male patients. * *p* < 0.05.

To identify age-specific BMI differences, patients were divided into eight age blocks for comparison (Figure 5). Females in the path-VF group had a significantly lower BMI compared to the control group at ages 50–59 (22.80 ± 5.12 (n = 3) vs. 31.87 ± 6.98 (n = 18), *p* < 0.001, *r* = 0.47, 70–79 (25.24 ± 4.22 (n = 28) vs. 28.74 ± 5.40 (n = 20), *p* = 0.021, *d* = 0.74) and 80+ (26.06 ± 4.90 (n = 25) vs. 31.34 ± 4.91 (n = 7), *p* = 0.031, *r* = 0.42). In males, a significantly lower BMI in the path-VF group was observed in the 70–79 (23.26 ± 3.60 (n = 5) vs. 28.41 ± 4.21 (n = 12), *p* = 0.018, *r* = 0.58) and 80+ age blocks (24.46 ± 3.59 (n = 13) vs. 27.26 ± 2.63 (n = 9), *p* = 0.048, *d* = 0.86) compared to the control group.

### 3.5. Binary Logistic Regression Analysis

Binary logistic regression analysis (Table 3) showed that age and BMI were significantly associated with the risk of path-VF. The female gender was associated with a higher risk, although this was not statistically significant (β = 0.73, *p* = 0.054), while a higher BMI was found to be significantly protective (β = −0.10, *p* = 0.03). Being of an advanced age also considerably increased this risk (β = 0.41, *p* < 0.001).

**Figure 6:**
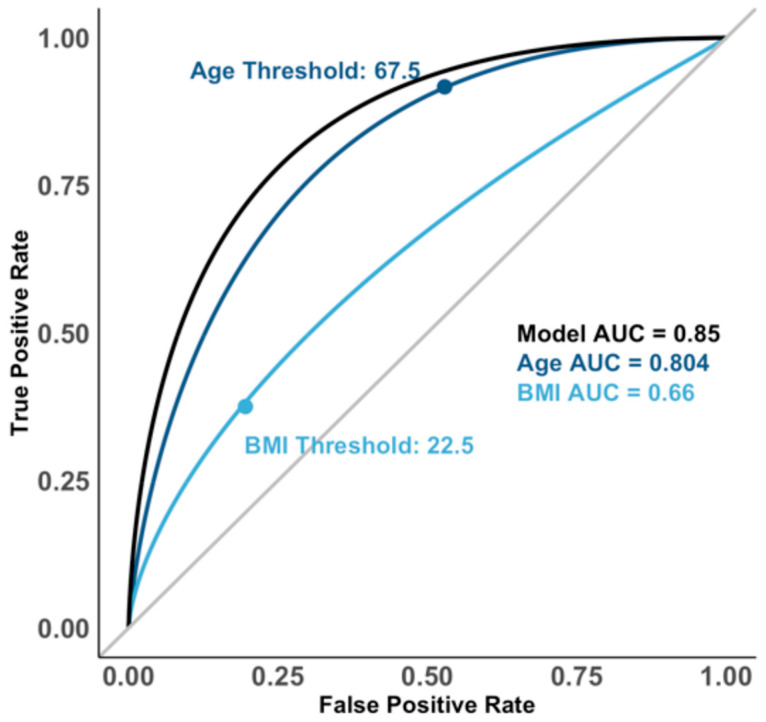
ROC curves of the logistic regression model with predictors of age and BMI for the risk of attending path-VF. AUC: area under the curve.

The ROC analysis (Figure 6) determined thresholds of 67.5 years for age (AUC = 0.804) and 22.5 kg/m^2^ for BMI (AUC = 0.66). The whole logistic regression model showed excellent predictive performance (AUC = 0.85).

**Figure 7:**
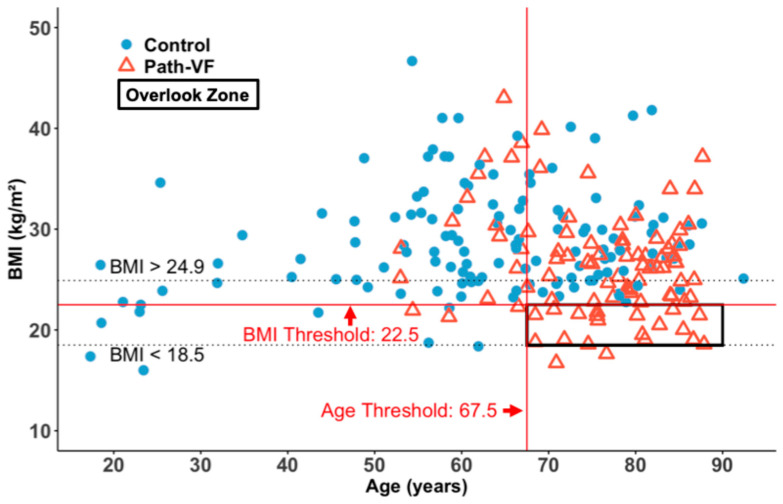
Age and BMI distribution highlighting the ‘overlook zone’ for the risk of path-VF. The area between the horizontal dashed lines represents the WHO-defined normal BMI range (18.5–24.9 kg/m^2^). The solid red line represents the BMI threshold of 22.5 kg/m^2^, and the solid vertical line indicates the age threshold of 67.5 years. The black frame marks the ‘Overlook Zone’, in which all patients over 67.5 years with a BMI of 18.5–24.9 kg/m^2^ showed path-VF.

Ultimately, all 225 patients were plotted on an age versus BMI scatterplot, highlighting the normal BMI range and thresholds for age and BMI (Figure 7). An ‘overlook zone’ was identified within the normal BMI range, consisting of 19 patients over 67.5 years, all of whom were in the path-VF group. Notably, no patients from the control group were found in this zone. These patients accounted for 20.2% (19/94) of the path-VF group and represented 24.7% (19/77) of all patients older than 67.5 years, emphasising a potential overlooked risk in ageing patients with a normal BMI.

## 4. Discussion

### 4.1. Protective Effects of Higher BMI in the Elderly

Recent evidence indicates that the relationship between BMI and health outcomes exhibits clear age-dependent patterns. Several studies suggest that in older adults, a BMI classified as “overweight” by WHO standards (>25 kg/m^2^) may not be detrimental and can even confer protective effects in specific clinical contexts. For example, Huang et al., in a large cohort of hypertensive patients over 45 years of age, found that a BMI of 25 to 29.9 kg/m^2^ was significantly associated with reduced all-cause mortality, whereas underweight (BMI < 18.5 kg/m^2^) increased early mortality risk [28]. Similarly, a meta-analysis by Winter et al. reported that in individuals aged ≥ 65 years, a BMI below 22 kg/m^2^ was associated with higher mortality, a trend not observed in younger populations [29]. Additionally, a retrospective analysis of 22,903 inpatients found that elderly individuals with a BMI > 25 kg/m^2^ had a lower in-hospital mortality rate and shorter lengths of stay [30]. Collectively, these findings suggest the need to re-evaluate the clinical interpretation of BMI in older populations, as the current WHO classification may underestimate the risks associated with a low BMI and the potential benefits of a higher BMI in ageing individuals.

### 4.2. Age-Related Decline in Spinal Bone Strength

Secondly, the relationship between ageing and bone strength deterioration is well established. Numerous studies have demonstrated that BMD declines progressively with age, with the spine being particularly susceptible to age-related osteoporotic changes. Kamei et al. identified age 50 as a critical point for accelerated bone loss in women, particularly in the thoracic and lumbar regions, indicating an early risk of spinal osteoporosis in this group [31]. Szulc et al. observed site-specific reductions in BMD among 1040 adult men, with a significant loss in the lumbar spine. This was particularly evident in individuals without advanced facet joint arthrosis, supporting the sensitivity of spinal bone loss to age [32]. Rondanelli et al. found that the prevalence of osteoporosis in the lumbar spine and hip increased substantially with age. Although BMI was positively correlated with BMD, this association became weaker in older adults [33]. These findings highlight age as a key factor influencing osteoporosis and vertebral bone loss.

### 4.3. Impact of Underweight and Obesity on Spine Surgery Risk

BMI exhibits a U-shaped relationship with surgical outcomes, indicating increased postoperative complications for both underweight and morbidly obese patients [6,34].

Obese patients are reported at higher risk for postoperative complications, such as spondylodiscitis [10], surgical site infections, prolonged hospital stays, and increased readmission rates compared to those with a normal BMI [6,12,35]. Biomechanically, obesity adds stress to vertebral structures and surgical implants, potentially causing hardware failure and adjacent-segment degeneration [11]. However, some studies indicate that the impact of overweight and moderate obesity on spinal surgery outcomes may not always be significant or consistent [5,36].

On the other hand, recent studies have also highlighted significant risks associated with underweight in spinal surgery outcomes [15,18]. Underweight patients often face complications such as delayed wound healing due to malnutrition, increased infection risk, and slowed postoperative recovery [5]. Additionally, patients with a lower BMI typically exhibit poorer bone microarchitecture quality, elevating their susceptibility to fractures and PSL after PISF [37], thereby negatively impacting both their short-term recovery and long-term quality of life.

However, these studies mainly applied the standard WHO BMI classification [9], focusing predominantly on abnormal BMI categories [5,38]. Detailed analyses within the normal BMI range, which represents 85% of BMI distributions [39], and comprehensive age-specific evaluations remain lacking, despite conflicting evidence regarding risks at BMI extremes [18,36,40].

### 4.4. Rethinking BMI Thresholds in Vertebral Fragility Assessment

In this study, we initially confirmed the validity of our grouping criteria by verifying significantly lower vertebral HU values in the path-VF group, aligning with previous reports that HU values are strongly correlated with decreasing BMD and reliably predict PSL [41].

Notably, our analysis revealed distinct associations between BMI categories and path-VF risk compared to studies in the existing literature, which generally links abnormal BMI with increased vertebral fragility risk [5,38,39]. Specifically, we observed an elevated risk among normal-weight females, contrasting with a protective effect seen in overweight males and obese individuals. These findings suggest that traditional BMI classification might inadequately capture the nuanced relationship between body weight and vertebral health, particularly within the normal-weight range among older adults.

Our further analysis across age cohorts revealed distinct age–BMI patterns between groups. Specifically, a decreased BMI with an advanced age correlated with an increased likelihood of path-VF, particularly among elderly males, whereas females consistently exhibited a lower BMI across age categories. Interestingly, despite lower BMI values in the path-VF group, the mean BMI consistently remained within the normal-weight range, implying that standard BMI thresholds may underestimate vertebral fragility risk in elderly patients. This pattern aligns with the broader geriatric literature, highlighting an elevated mortality risk associated with a low/normal BMI in older populations [19].

A binary logistic regression reinforced these associations, indicating that an advanced age and the female gender independently increased the path-VF risk, while a higher BMI conferred protection specifically regarding path-VF, though not necessarily indicating overall health benefits. Indeed, overweight and obesity remain well-established risk factors for numerous perioperative and systemic complications [42]. The protective effect observed here could be explained by factors such as enhanced mechanical support from increased body mass and better overall nutritional status, which may contribute positively to bone quality [43,44].

Finally, our findings suggest a clinically relevant subgroup of elderly patients who, despite having a normal BMI, are at elevated risk for path-VF and could easily be overlooked under current WHO guidelines. This highlights the importance of re-evaluating BMI thresholds or supplementing BMI assessments with additional evaluations for bone fragility when planning spinal surgeries in older populations.

## 5. Limitations

This study has several limitations, starting with its single-centre design in one West-ern European country, which means the findings might reflect specific local surgical practices and patient profiles, potentially limiting their generalizability to other populations or healthcare systems. Furthermore, the absence of data on comorbidities, nutritional status, and various musculoskeletal indicators may have restricted a comprehensive analysis of all factors contributing to vertebral fragility. Finally, the 22.5 kg/m^2^ BMI threshold, though derived from an ethnically diverse single-centre cohort, requires external validation through larger, population-specific studies to confirm its broader applicability.

## 6. Conclusions

Our findings suggest that the current WHO-defined lower limit of a normal BMI (18.5 kg/m^2^) may be insufficient for assessing the risk of path-VF in patients older than 67.5 years. Relying solely on this threshold may result in the missed identification of approximately 24.7% of elderly patients at risk of early pedicle screw loosening due to path-VF. While we do not propose modifying the BMI classification itself, we recommend that spinal surgeons interpret BMI with greater caution in this population. Even patients classified as normal weight may require further evaluation for compromised bone integrity if their BMI lies at the lower end of the normal range.

## Figures and Tables

**Table 1:** Demographics of the involved patients.

	Age				
	n	Min.	Mean	Max.	SD
Total	225	17	68.11	92	15.16
Male	88	17	65.26	92	17.78
Female	137	23	69.95	87	12.94
The path-VF group	94				
Male	26	53	75.72	87.4	9.83
Female	68	52	75.85	87	8.27
The control group	131				
Male	62	17	60.00	92	18.57
Female	69	23	64.13	86	14.09
Balloon Kyphoplasty (exclusive tumour)	33	58	78.27	87	8.68
1st PISF with reinforcement	47	53	74.77	86	8.21
1st PISF without reinforcement	141	17	63.38	92	16.25
2nd PISF due to PSL within six months	29	52	71.80	84	9.19

Path-VF: pathological vertebral fragility. PISF: posterior instrumented spinal fusion; PSL: pedicle screw loosening.

**Table 2:** BMI classification by gender and group.

Gender	BMI-Classification	Control (n)	Path-VF (n)	Total (n)
Female	Obesity	28	12	40
	Overweight	23	26	49
	Normal weight	16	29	45
	Underweight	2	1	3
Male	Obesity	17	6	23
	Overweight	28	8	36
	Normal weight	16	11	27
	Underweight	1	1	2

**Table 3:** Logistic regression for the risk of path-VF.

Predictor:	β	SE β	z Value	*p*
Constant	−5.03	1.20	−4.19	2.77 × 10^−5^ ***
Gender (Female)	0.73	0.38	1.93	0.054
Age	0.41	0.08	4.84	1.29 × 10^−6^ ***
BMI	−0.10	0.04	−2.17	0.03 *

SE: standard error; *: *p* < 0.05; ***: *p* < 0.001.

## Data Availability

The datasets generated and/or analysed during the current study are not publicly available due to institutional data ownership. The data are the property of RKH Orthopaedic Clinic Markgröningen, 71706 Markgröningen, Baden-Württemberg, Germany. Data may be made available upon reasonable request and with prior approval from the institution. Requests should be directed to the corresponding author.

## Abbreviations

The following abbreviations are used in this manuscript:

PSL	Pedicle screw loosening
PISF	Posterior instrumented spinal fusion
VF	Vertebral fragility
Path-VF	Pathological vertebral fragility
WHO	World Health Organization
BMI	Body mass index
HU	Hounsfield units
OR	Odds ratio
BMD	Bone mineral density
SD	Standard deviation
SE	Standard error
CI	Confidence intervals
ROC	Receiver operating characteristic curve
AUC	Area under the curve
*d*	Cohen’s d
*r*	Rank-biserial correlation coefficient
*r_p_*	Pearson’s correlation test coefficient
*q*	Fisher’s Z-test coefficient Cohen’s q
